# Multicellular Tumor Spheroids as a Model for Assessing Delivery of Oligonucleotides in Three Dimensions

**DOI:** 10.1038/mtna.2014.5

**Published:** 2014-03-11

**Authors:** Kyle Carver, Xin Ming, Rudolph L Juliano

**Affiliations:** 1 Division of Molecular Pharmaceutics, UNC Eshelman School of Pharmacy, University of North Carolina, Chapel Hill, North Carolina, USA

## Abstract

Oligonucleotides have shown promise in selectively manipulating gene expression *in vitro*, but that success has not translated to the clinic for cancer therapy. A potential reason for this is that cells behave differently in monolayer than in the three-dimensional tumor, resulting in limited penetration and distribution of oligonucleotides in the tumor. This may be especially true when oligonucleotides are associated with nanocarriers such as lipoplexes and polyplexes, commonly used delivery vehicles for oligonucleotides. The multicellular tumor spheroid (MCTS), a three-dimensional model that closely resembles small avascular tumors and micrometastases, has been utilized as an intermediate between monolayer culture and *in vivo* studies for the screening of small-molecule drugs. However, spheroids have been little used for the study of various oligonucleotide delivery formulations. Here, we have evaluated the uptake and efficacy of splice-switching antisense oligonucleotides using various delivery modalities in two- and three-dimensional culture models. We find that the size of the delivery agent dramatically influences penetration into the spheroid and thus the biological effect of the oligonucleotides. We hypothesize that the MCTS model will prove to be a useful tool in the future development of oligonucleotide delivery formulations.

## Introduction

Oligonucleotides can modulate the expression of genes in a highly selective manner and thus may provide an important approach to therapy for cancer and other diseases where current therapeutic modalities lead to unwanted side effects.^1^ Delivery of oligonucleotides *in vitro* is commonly achieved through the use of complexation with cationic lipoplexes or polyplexes^2,3^ although other potential delivery strategies have also been investigated.^4,5^ However, the many biological barriers *in vivo* present a challenge for oligonucleotide delivery to their sites of action within cells of interest.^6,7^

Current nanocarrier delivery systems can passively target and accumulate in some tumors through the enhanced permeation and retention effect caused by leaky tumor vasculature^8^ but also tend to diffuse poorly throughout the interstitial space of a tumor.^9^ The result is that drugs localize in regions proximal to blood vessels, leaving distal regions of the tumor untreated.^10,11^ Therefore, although oligonucleotide delivery systems can concentrate in the tumor, there may not be sufficient penetration and distribution throughout the tissue to produce a therapeutic effect.

Even though poor penetration into tumors may be a contributing factor to inadequate therapeutic effects of nanoparticle delivery systems,^10^ such systems are commonly tested *in vitro* using monolayer cultures where penetration is unnecessary. Cells grown in two dimensions lack many of the characteristics of those found in tumors, including a nutritional gradient, a complex microenvironment, and an altered genetic profile.^12,13^ Furthermore, the nature of tensional forces produced in three dimensions affects how cells behave compared with monolayer cultures.^14^

The multicellular tumor spheroid (MCTS) model compensates for many of the deficiencies seen in monolayer cultures. Spheroids on the scale of 200–500 µm develop chemical gradients of oxygen, nutrients, and catabolites, while having morphological and functional features similar to tumors.^15,16^ Therefore, assays utilizing the MCTS model allow for the assessment of drug penetration and are more predictive of *in vivo* success compared with monolayer cultures.^16,17^ There is, however, a limited amount of information on delivery and effects of oligonucleotides using the MCTS model.

Evaluation of various strategies for oligonucleotide delivery into tumors in animals is a challenging, expensive, and time-consuming process. We suggest that the tumor spheroid model may provide a useful, convenient, and rapid model for testing and understanding oligonucleotide delivery approaches similar to its value for testing conventional anticancer drugs. Here, we examine the behavior of several oligonucleotide delivery approaches in tumor spheroids versus conventional tissue culture. In particular, we evaluate molecular-scale arginine-glycine-aspartic acid (RGD)–oligonucleotide conjugates versus the same conjugates in nanoscale complexes with cationic lipids or polymers. We also examine the behavior of a RGD–oligonucleotide–albumin conjugate having macromolecular dimensions.

## Results

### MCTS characterization

**Figure 1:**
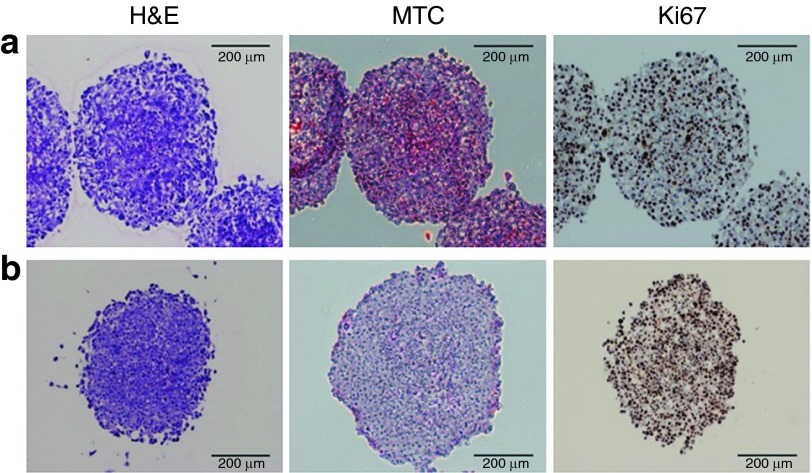
**Characterization of spheroid morphology.** Spheroids generated from (**a**) A375 GFP654 and (**b**) A375 Luc705 cell lines were fixed and sectioned prior to being stained with hematoxylin and eosin (H&E), Masson's trichrome (MTC), or Ki67. Bar = 200 µm. GFP, green fluorescent protein.

It has been suggested that some studies claiming to utilize MCTS were actually performed in nonspheroid loose aggregates,^16^ which do not establish the proper characteristics for testing drug penetration properties.^18^ Therefore, we first characterized the formation of spheroids in our system. Histological stains, hematoxylin and eosin (H&E) and Masson's trichrome, showed that the spheroids grow to ~400 µm in diameter and are comprised of very densely packed cells; however, they lack substantial collagen deposits (**Figure 1a**,**b**). In agreement with previous reports on MCTS of this approximate size, a homogenously positive Ki67 stain demonstrated that there is no quiescent core of cells. Based on these observations, A375 cells form valid spheroids for the MCTS model and represent a potentially useful *in vitro* model for early-stage, avascular tumors as well as micrometastasis.

### Comparison of monolayer and MCTS uptake

**Figure 2:**
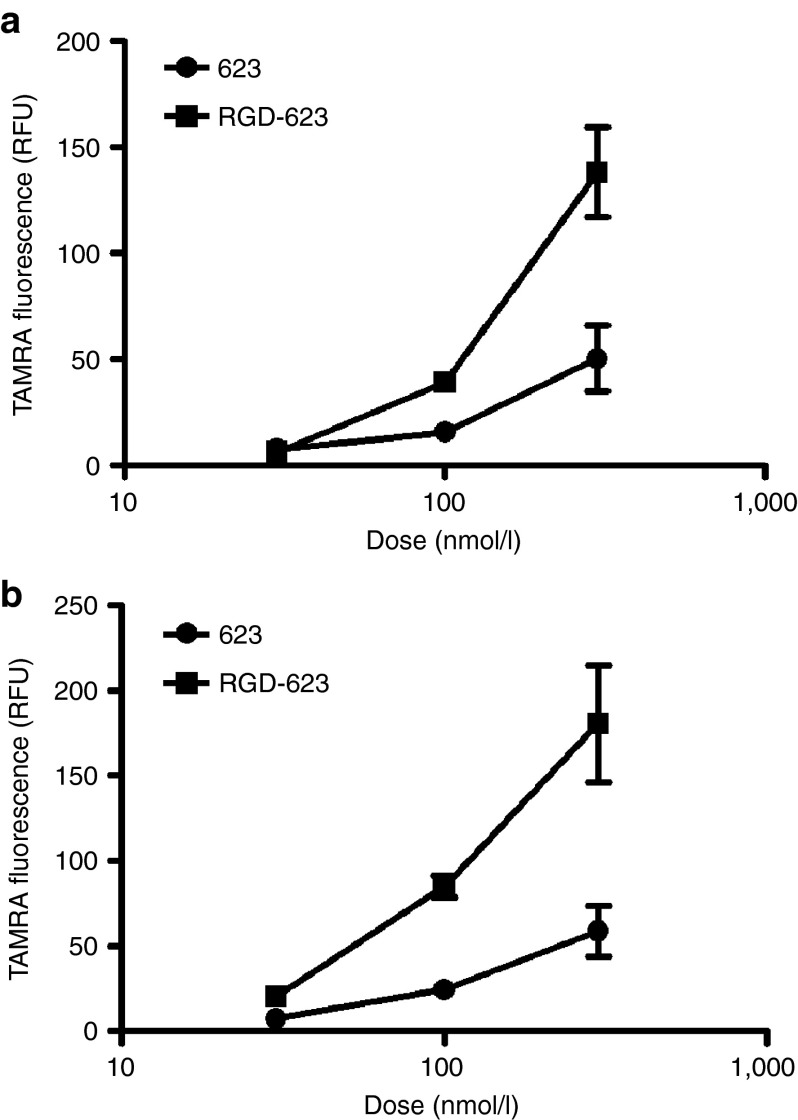
**Cellular uptake of unmodified 623 and RGD-623.** (**a**) A375 GFP654 and (**b**) A375 Luc705 spheroids were treated with various doses either unmodified 623-TAMRA or RGD-623-TAMRA splice-switching oligonucleotide for 16 hours. After dispersion of the spheroids, TAMRA fluorescence was measured by flow cytometry and quantified as cellular uptake as mean ± SEM. *n* = 3. GFP, green fluorescent protein; RFU, relative fluorescence unit; RGD, arginine-glycine-aspartic acid.

To begin to evaluate the transport properties of spheroids, we simply examined the uptake of TAMRA-labeled 623 or its RGD conjugate (**Figure 2a**,**b**). As seen, the uptake of 623 or RGD-623 increased progressively with dose with higher levels of uptake being attained with the RGD-623 conjugate, in agreement with a previous work in monolayer culture.^19^ For the remainder of our studies, we decided to work with the RGD-623 conjugate.

**Figure 3:**
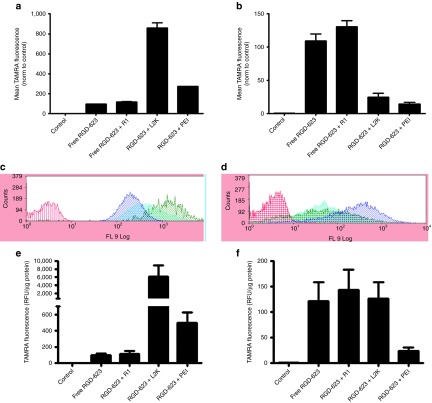
**Differential cellular uptake in two- and three-dimensional culture.** A375 GFP654 cells grown in (**a**) monolayer or (**b**) spheroid culture were treated with 100 nmol/l in the specified delivery formulation for 16 hours. Cellular uptake was quantified as TAMRA fluorescence measured by flow cytometry as mean ± SEM. *n* = 3–10. (**c**,**d**) Representative flow cytometry histograms of monolayer and spheroid uptake, respectively. Red, control; blue, RGD-623; green, RGD-623 L2K; teal, RGD-623 polyethylenimine (PEI). HeLa Luc705 cells grown in (**e**) monolayer or (**f**) spheroid were treated with 100 nmol/l of the specified delivery formulation of the RGD-623-TAMRA for 16 hours. Cellular uptake was quantified as TAMRA fluorescence normalized to protein content measured on a FLUOstar Omega plate reader as mean ± SEM. *n* = 3–7. In some cases, Retro-1 was added at 100 µmol/l for 2 hours. GFP, green fluorescent protein; RFU, relative fluorescence unit; RGD, arginine-glycine-aspartic acid.

One of the reasons that therapeutics screened in monolayer cultures have reduced efficacy *in vivo* is their failure to effectively penetrate and distribute throughout the tissue.^10^ In addition, as the size of a delivery formulation increases, penetration through the tumor decreases.^20,21^ The attenuation of penetration may be due to limited intercellular space and cell surface area when compared with monolayer culture. To test whether the MCTS model can effectively differentiate delivery formulations of different sizes, we treated cells grown in both monolayer and spheroid conditions and measured cellular uptake of a TAMRA fluorophore-conjugated splice-switching oligonucleotides (SSOs). Uptake of oligonucleotides via cationic lipid complexes (mean particle size = 870 nm) or polyplexes (polyethylenimine) (mean particle size = 336 nm),^22^ in both A375 and HeLa cells, was substantially higher than free oligonucleotides in monolayer culture (**Figure 3a**,**e**).

When used in three-dimensional spheroid culture, the larger oligonucleotide formulations displayed significantly reduced delivery compared with their two-dimensional levels, as well as to the uptake of free RGD-623 oligonucleotides (**Figure 3b**,**f**). The profile of the flow cytometry histograms of TAMRA fluorescence also shows that the distribution of the complexed oligonucleotides was very heterogeneous in spheroids compared with monolayer culture, with values ranging over three decades (**Figure 3c**,**d**). Interestingly, there was little difference in free RGD-623 oligonucleotide uptake between monolayer and spheroid cultures, suggesting that the conjugated SSO was able to penetrate into the MCTS. The unconjugated SSO, 623, was also able to diffuse throughout the spheroid (data not shown), suggesting that the result is more due to the size of the oligonucleotides rather than specific binding via RGD. We also examined the effect of serum on the uptake process. As seen in **Supplementary Figure S1**, the inclusion of 10% serum produced only a very modest reduction in uptake of the RGD-623 conjugate by the spheroids.

Because uptake of oligonucleotides initially leads to sequestration within endosomal compartments, we chose to also test the small-molecule Retro-1, which we have previously shown to improve oligonucleotide release from endosomes in monolayer culture.^23^ To confirm that Retro-1 works on endosome stability rather than uptake, we examined its effects on the accumulation of oligonucleotides. Retro-1 had no effect on uptake of RGD-623 in either monolayer or MCTS cultures (**Figure 3**).

**Figure 4:**
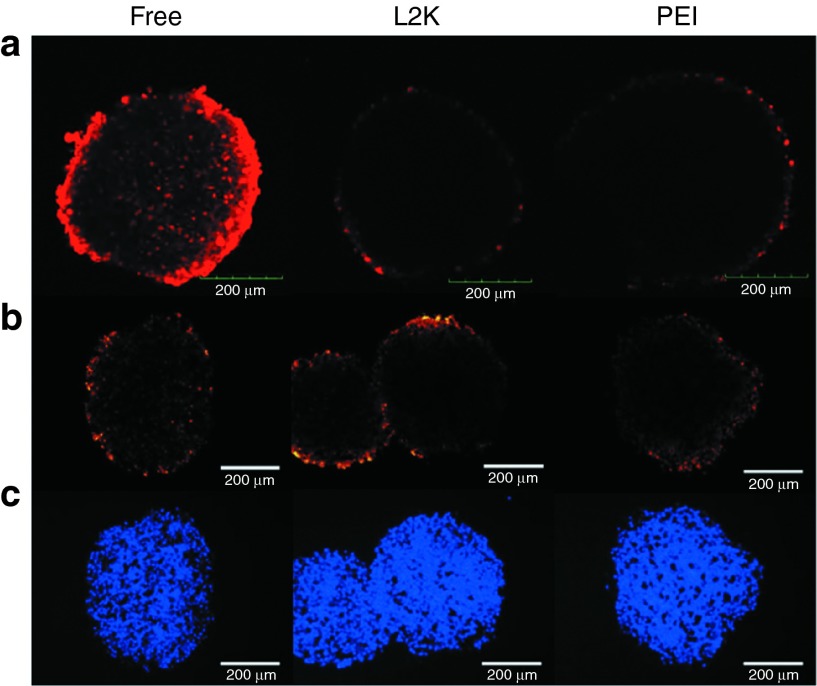
**Comparison of delivery formulation penetration in spheroids.** Spheroids were treated with 100 nmol/l of the oligonucleotides using the specified delivery method (free, lipoplex (L2K), or polyplex (polyethylenimine (PEI))) for 16 hours and then fixed and (**a**) mounted for confocal microscopy or (**b**) sectioned for wide field fluorescence microscopy. (**c**) 4′,6-diamidino-2-phenylindol fluorescence depicting the density of cells within the section spheroids. Confocal image is at a depth of 70 µm, wide field section is at a depth of ~80 µm. Bar = 200 µm. GFP, green fluorescent protein.

To further assess the uptake and distribution of the various delivery formulations in MCTS, we imaged spheroids using confocal microscopy (**Figure 4a**). At a penetration depth of 70 µm, the limit of the laser, the cationic lipid or polymer complexed oligonucleotides were only able to accumulate in cells at the exterior of the spheroid as depicted by the ring of fluorescence. Uptake of the free oligonucleotides, on the other hand, was variable from cell-to-cell but was distributed throughout the spheroid. Differences between the delivery formulations observed by confocal microscopy were confirmed by physically sectioning the spheroids and imaging TAMRA fluoresence (**Figure 4b**) at a section ~80-µm deep and using a 4′,6-diamidino-2-phenylindol counterstain to demonstrate the cell density of spheroids at the given depth (**Figure 4c**).

### Comparison of splice-switching efficacy

**Figure 5:**
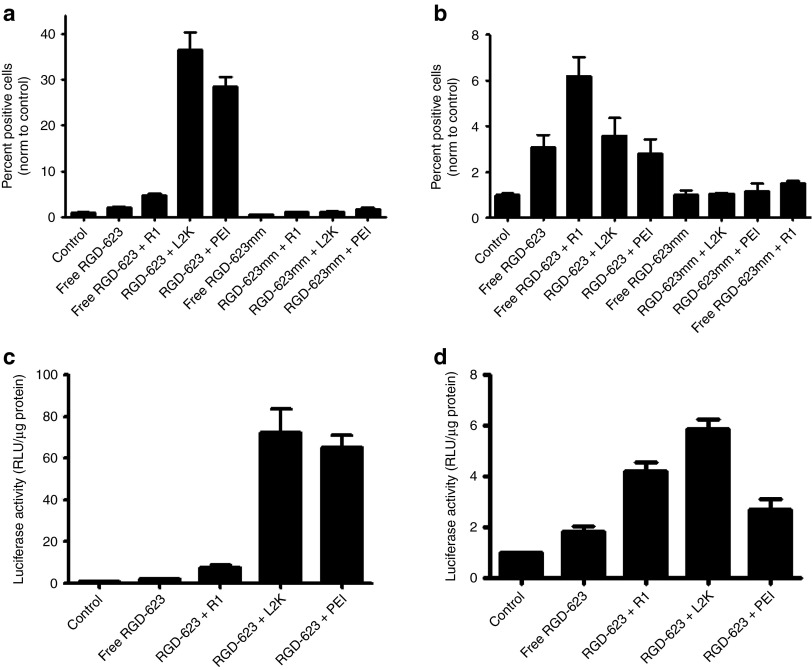
**Differential efficacy of delivery formulations in monolayer and spheroid culture.** A375 GFP654 cells were treated in (**a**) monolayer and (**b**) spheroid culture with 100 nmol/l of the oligonucleotides using the specified delivery method for 16 hours and further incubated for 48 hours. Cells were then fixed and GFP expression was measured using flow cytometry. Quantification is expressed as a percent of GFP-positive cells after normalization to control, depicted as mean ± SEM. *n* = 5–10. HeLa Luc705 cells were treated in (**c**) monolayer and (**d**) spheroid culture with 100 nmol/l of the oligonucleotides using the specified formulation for 16 hours and further incubated for 48 hours. Cells were lysed, and luciferase activity was measured on a FLUOstar Omega plate reader. Quantification is expressed as relative luminescence units per µg protein after normalization to control, depicted as mean ± SEM. *n* = 3–5. HSA, human serum albumin; GFP, green fluorescent protein; RGD, arginine-glycine-aspartic acid.

We further sought to assess how oligonucleotide efficacy differs between two- and three-dimensional cell cultures. Cationic delivery formulations not only enhance the uptake of oligonucleotides in monolayer cultures but also improve delivery to the site of action within the cell. Oligonucleotides entering the cell via free uptake mechanisms remain sequestered within endosomes, resulting in a limited biological effect.^24^ As expected, the cationic complexes significantly increased the induction of splice correction of both the green fluorescent protein 654 (GFP654) and Luc705 reporter genes in cells grown in monolayer culture, whereas free oligonucleotide induction levels were only slightly higher than untreated controls (**Figure 5a**,**c**). The addition of Retro-1 to free uptake samples resulted in a significant increase in splice correction as we have reported previously.^23^ A RGD-623 mismatch oligonucleotide was used in GFP654 cells to eliminate the possibility that the delivery formulations were having a direct effect on splice switching.

**Figure 6:**
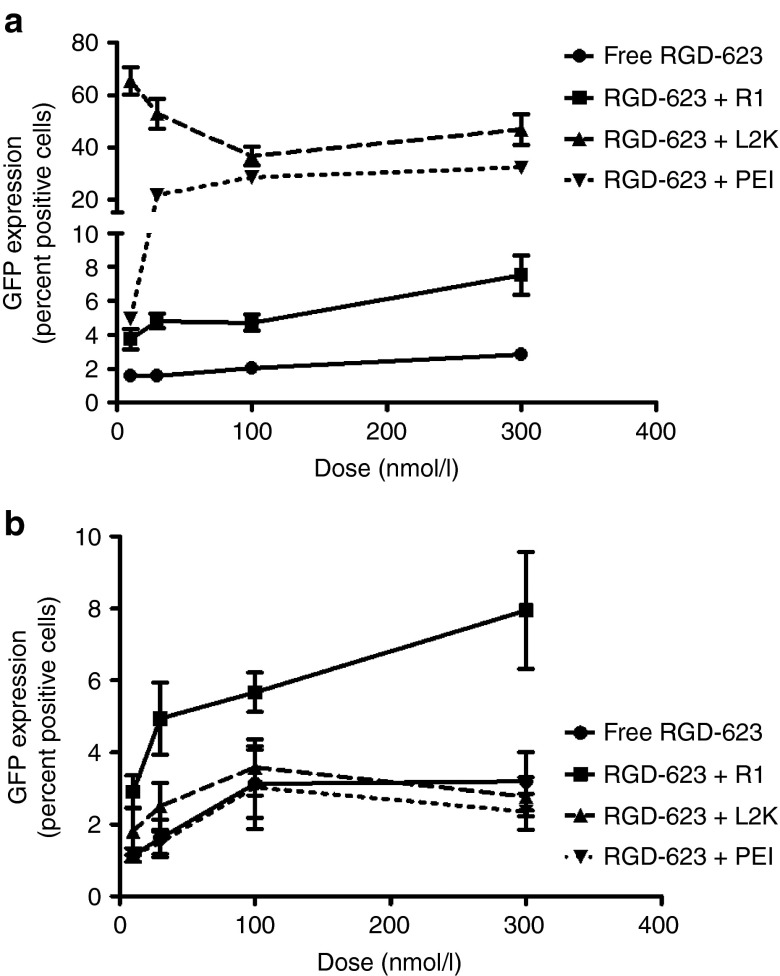
**Dose response in monolayer and spheroid cultures.** A375 GFP654 cells were treated by varying doses of oligonucleotide formulations in (**a**) monolayer and (**b**) spheroid cultures as mentioned in **Figure 5**. Depicted as mean ± SEM. *n* = 3–6. HSA, human serum albumin; GFP, green fluorescent protein; RGD, arginine-glycine-aspartic acid.

In MCTS, cationic lipid and polyplex oligonucleotide complexes displayed significantly reduced splice switching compared with their effects in monolayer culture (**Figure 5b**,**d**). This result is most likely due to the limited penetration of the complexed oligonucleotides in spheroids. Furthermore, the lipoplexes exhibit a negative zeta potential, whereas the polyplex charge is positive,^22^ suggesting that any reduction in penetration is most likely due to the size of the complex rather than charge effects. In A375 GFP654 spheroids, the greater penetration and distribution of free RGD-623 throughout the spheroid seems to compensate for the poor release from endosomes, resulting in equivalent induction compared with complexed oligonucleotides across all doses investigated (**Figure 6a**,**b**). Enhancing the endosomal release of RGD-623, through incubation with Retro-1, significantly improved the induction seen in both spheroid types. In HeLa Luc705 cells, induction through Lipofectamine 2000 was still greater than free RGD-623 most likely due to the fact that luciferase measures total induction instead of a percentage of affected cells (**Figure 5**). Therefore, large induction at the periphery of the spheroid most likely drove up the overall induction measured. Regardless, there is a remarkable attenuation of induction between monolayer and spheroid cultures for the cationic complexes that is similar for both cell lines.

### Small oligonucleotide conjugates

The use of Lipofectamine 2000 and jetPEI complexes demonstrate how delivery differs between two- and three-dimensional cultures. However, these complexes are larger than many delivery vehicles currently under investigation. In order to further evaluate the MCTS model, we also assessed the delivery of an intermediate-sized delivery moiety. This is comprised of RGD-623 oligonucleotides conjugated to human serum albumin (HSA), as recently developed in our laboratory.^25^ These small conjugates are ~13 nm in size and therefore represent a size at the lower range of nanoparticles.

**Figure 7:**
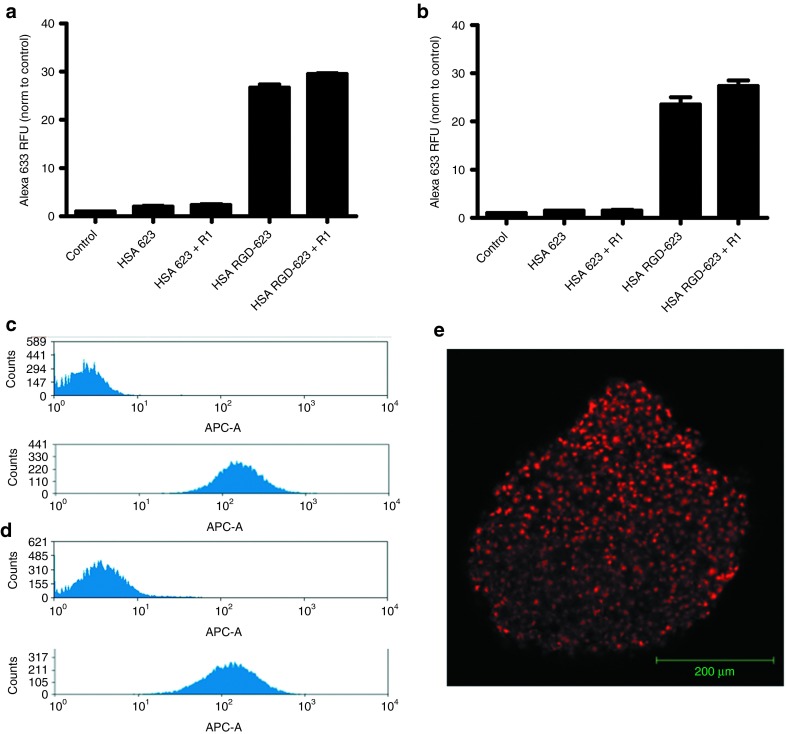
**Cellular uptake of human serum albumin (HSA)**–**PMO conjugates.** A375 GFP654 cells grown in (**a**) monolayer or (**b**) spheroid culture were treated with 100 nmol/l HSA–PMO conjugate with and without conjugated RGD peptides for 16 hours. In some cases, Retro-1 was added at 100 µmol/l for 2 hours. Cellular uptake was measured as Alexa633 fluorescence by flow cytometry and normalized to control, depicted as mean ± SEM. *n* = 5–10. Representative flow cytometry histograms of treatment with 100 nmol/l HSA–RGD conjugates in (**c**) monolayer and (**d**) spheroid culture. (**e**) Confocal image of HSA–RGD-623 conjugate in multicellular tumor spheroid. GFP, green fluorescent protein; PMO, phosphorodiamidate morpholino; RFU, relative fluorescence unit; RGD, arginine-glycine-aspartic acid.

**Figure 8:**
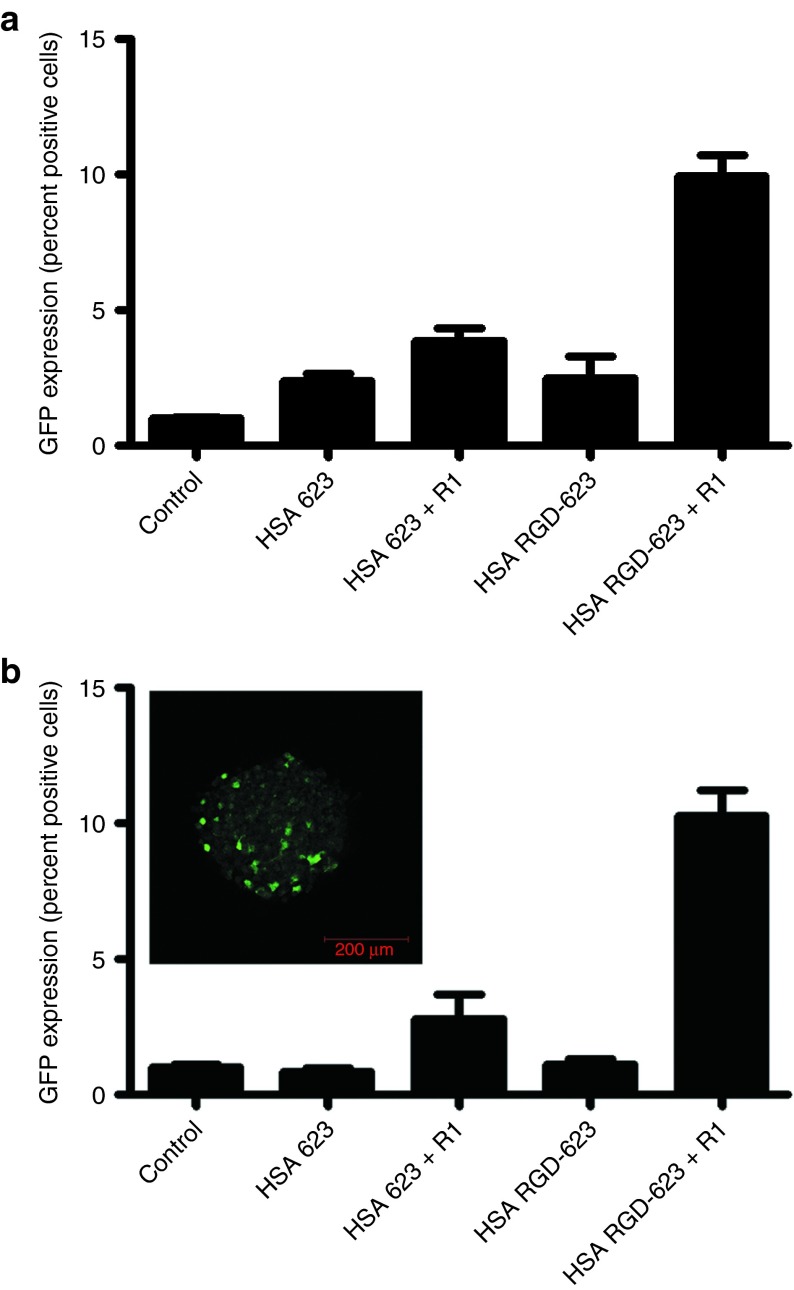
**Induction of GFP by human serum albumin (HSA)**–**PMO conjugates.** A375 GFP654 cells grown in (**a**) monolayer or (**b**) spheroid culture were treated with 100 nmol/l HSA–PMO conjugate with and without conjugated RGD peptides for 16 hours followed by further 48-hour incubation. In some cases, Retro-1 was added at 100 µmol/l for 2 hours. Cells were fixed, and GFP expression was measured using flow cytometry and quantified as a percentage of induced cells after normalization to control, depicted as mean ± SEM. *n* = 3–10. Inset: confocal image of representative spheroid treated with HSA–RGD-623 and Retro-1. Bar = 200 µm. GFP, green fluorescent protein; PMO, phosphorodiamidate morpholino; RGD, arginine-glycine-aspartic acid.

Interestingly, as seen in **Figure 7a**,**b**, there is no significant difference in cellular uptake between monolayer and spheroid culture. In fact, examination of the flow cytometry histograms shows that there is a unimodal peak of similar mean value and distribution pattern in both culture conditions, which suggests that the HSA–RGD-623 conjugate is able to penetrate and diffuse throughout the spheroid (**Figure 7c**,**d**). These observations were confirmed with confocal microscopy (**Figure 7e**). Furthermore, when we tested the efficacy of the HSA–RGD-623 conjugate in GFP654 cells, there was no loss of splice-switching induction between monolayer and spheroid culture (**Figure 8**). Confocal microscopy of a HSA–RGD-623– and Retro-1–treated spheroid demonstrates that the induction of GFP was not simply on the periphery.

Taken together, these data suggest that the MCTS model is able to differentiate between various oligonucleotide delivery formulations. The large complexes fail to penetrate through the spheroids, whereas the smaller conjugates are able to effectively diffuse through the interstitial space of the spheroids. Conjugation of RGD peptides to the 623 oligonucleotides enhanced cellular uptake without leading to a binding site barrier effect. MCTS, therefore, may provide a useful intermediate step that can screen oligonucleotide delivery methods before proceeding to *in vivo* experiments.

## Discussion

Oligonucleotides can modulate gene expression in tumor cells in monolayer cultures. However, in order to elicit these effects, a delivery system such as cationic lipoplexes or polyplexes is usually required. The unique nature of the tumor microenvironment, including densely packed cells, poses additional biological barriers that are challenges for oligonucleotide delivery.^26^ Therefore, the development of next-generation delivery modalities must be tested against these barriers. Although xenograft tumors have been shown to effectively mimic clinical responses to drugs,^27,28^ the high animal burden, cost, and difficulty in performing xenograft models limit their use to late-stage oligonucleotide delivery candidates. Therefore, an *in vitro* culture system that more accurately models tumors than monolayer cultures would be beneficial and cost effective for early-stage and high-throughput screening of oligonucleotide delivery formulations.

The MCTS is one of the most widely characterized three-dimensional culture systems and has been shown to resemble small avascular tumors and micrometastasis.^17^ Much interest has been directed at MCTS for the screening of small-molecule drugs,^12,26,29,30^ and the model has been shown to mimic *in vivo* drug efficacy.^31,32^ However, there are limited data on the usefulness of spheroids for the assessment of oligonucleotide delivery formulations.^33^

In this study, we aimed to evaluate the utility of the MCTS model for testing oligonucleotide delivery strategies. Because cationic lipoplexes and polyplexes are the most widely used delivery methods in monolayer cultures, we initially tested their efficacy in spheroids. It is well known that while nanocarriers tend to concentrate in tumors due to enhanced permeation and retention effect, they sometimes fail to adequately penetrate through the tumor interstitial space.^11^ As would be expected due to their large size, we demonstrate that the delivery of oligonucleotides with the Lipofectamine 2000 lipoplex and jetPEI polyplex formulations is significantly attenuated in MCTS when compared with monolayer cultures. The large complexes can only deliver their payloads to the exterior cells of the spheroid, limiting both the percentage of cells induced (GFP654) and total induction (Luc705) compared with that in monolayer cultures.

Compared with complexed oligonucleotides, uptake of “free” RGD-623 in two dimensions is small as is the overall induction of splice switching. The limited induction results from both a lower total uptake of oligonucleotides and entrapment within the endosomal compartments of the cell. As we have shown previously, the small-molecule Retro-1 is able to significantly enhance the induction efficacy of SSOs by promoting release from endosomes.^23^ Interestingly, the uptake and induction profile of free oligonucleotides as well as the enhancement by Retro-1 was similar between monolayer culture and spheroids. These results suggest that molecular-scale RGD–oligonucleotide conjugates are not affected by the more complex nature of the spheroid. Although we did not test this directly, we anticipate that other types of oligonucleotides such as small interfering RNA or micro RNA antagomirs that are in the same size range as our SSOs would behave similarly in the spheroid context.

To further test the discrimination of delivery methods in two- and three-dimensional culture, we utilized a small (13 nm) nanoconjugate, developed in our laboratory,^25^ of HSA containing, on average, 10 RGD-623 phosphorodiamidate morpholino oligonucleotides. The ultra-nanoscale conjugate was able to penetrate homogenously throughout the tumor spheroid, as demonstrated by the unimodal peak of the flow cytometry histogram, and uptake levels were equivalent to those seen in monolayer. Furthermore, the splice-switching induction profile showed that a similar percentage of cells were induced in two- and three-dimensional culture.

Our studies suggest that MCTSs show a strong size discrimination for oligonucleotide carriers. To the extent that tumor spheroids accurately represent the behavior of tumors *in vivo*, our data suggest that small multivalent nanoconjugates of macromolecular scale may be most effective in delivering oligonucleotides to the interior of small avascular tumors or in directing therapy to micrometastases. However, a follow-up study investigating the efficacy of these various delivery formulations in xenograft or orthotopic tumors is necessary to fully validate the utility of MCTSs in this area.

## Materials and methods

*Cell culture.* A375 human melanoma cells were stably transfected with firefly luciferase (A375 Luc705)^19^ or GFP (A375 GFP654)^22^ expression cassettes containing a mutated intron. HeLa human cervical cancer cells (HeLa Luc705) were stably transfected with the firefly luciferase cassette.^34^ The mutated introns can be removed by using an SSO to alter splicing, thus giving inducible expression of the reporter, as described previously.^35^ All cells were grown in high-glucose Dulbecco's modified Eagle's medium (DMEM) (Sigma, St. Louis, MO) supplemented with 10% fetal bovine serum (FBS).

*Generation of MCTSs.* A375 GFP654, A375 Luc705, and HeLa Luc705 MCTSs were generated using the hanging drop method.^36^ Briefly, cells were trypsinized and resuspended in 20% FBS high-glucose DMEM at a concentration to achieve 8,000 cells per 30 µl (A375 GFP654) or 2,500 cells per 30 µl (A375 Luc705 and HeLa Luc705). In a 72-microwell plate (Nunc 438733; Thermo Fisher Scientific, Waltham, MA), 30 µl of culture medium with cells was added to each well; plates were then inverted and incubated at 37 °C for 3–5 days on an orbital shaker. Once formed, MCTSs, 10 spheroids per well, were transferred to a 48-well plate coated with 1.5% agarose for treatment.

*Formulation and cell treatment.* The SSO623 (5′-GTTATTCTTTAGAATGGTGC-3′), which can correct the mutated introns in GFP654 and Luc705 constructs and induce reporter gene expression, or its mismatch control, were used in all experiments.^19^ Complexes of oligonucleotides with a cationic lipid (Lipofectamine 2000; Life Technologies, Grand Island, NY) or cationic polymer (jetPEI; Polyplus, Illkirch, France) were prepared per the vendor's instructions. Conjugation of RGD (Arg-Gly-Asp) peptide to SSO623 phosphorothioate to form RGD-623,^19^ or SSO623 morpholino (phosphorodiamidate morpholino) to RGD and then to HSA (HSA-RGD-623),^25^ were performed as described previously. In both monolayer and MCTS, “free” oligonucleotide, HSA conjugate, and polyethylenimine polyplex uptake was performed in OptiMEM (Gibco, Life Technologies) media, whereas Lipofectamine 2000 uptake was done in 10% FBS DMEM to limit cellular toxicity. All groups were treated for 16 hours. In some cases, Retro-1, a small molecule that has been shown to enhance release of oligonucleotides from endosome compartments, was directly added to the wells at the end of the treatment period as previously described.^23^ Cells were further incubated for 2 hours before being washed and were incubated for an additional 48 hours in their native growth media (10% FBS DMEM for monolayer and 20% FBS DMEM for MCTS) before being harvested for analysis.

*Luciferase assay.* Cells were washed with phosphate-buffered saline before being digested with luciferase lysis buffer (New England Biolabs, Ipswich, MA) at a dilution of 1:4. After centrifugation, 50 µl of luciferin substrate (Promega, Madison, WI) was added to 10 µl of lysate, and luciferase activity was measured in a plate reader (FLUOstar Omega; BMG Labtech, Cary, NC) over a 5-second window. The sum of blank corrected data over seconds 2–5 of the window was used to quantitate induction. Induction was normalized to protein concentration measured with a bovine serum albumin assay (Thermo Scientific).

*Flow cytometry.* Cells were trypsinized and fixed in single cell suspension with 4% paraformaldehyde before being resuspended in phosphate-buffered saline for flow cytometry on an LSR II using a 488-nm laser with 510/20 band pass filter (GFP), 561-nm laser with 610/20 band pass (TAMRA), and 639-nm laser with 675/20 band pass (Alexa 633). After gating for live singlet cells, uptake was quantified by normalizing mean TAMRA fluorescence value with the control cell's autofluorescence. GFP654 induction was quantified by gating for cells expressing a positive level of fluorescence based on untreated cells and then normalizing to control levels.

*Histology.* MCTSs were harvested and fixed in 4% paraformaldehyde before being cryoprotected in 30% sucrose / phosphate-buffered saline. Up to 10 spheroids were placed in a 1.5% agarose block in order to maintain orientation before being embedded in optimal cutting temperature compound (Sakura, Torrance, CA) and sectioned. Serial slides were then processed with hematoxylin and eosin to demonstrate the structure and cell density of MCTS, Masson's trichrome to demonstrate cell density and collagen content within the MCTS, a Ki67 antibody (Vector Laboratories, Burlingame, CA) to show cellular proliferation throughout the MCTS, or mounted with 4′,6-diamidino-2-phenylindol for fluorescent imaging. An Olympus IX71 microscope with 10× objective and cellsans entry software was used to collect images of the spheroid sections. Scale bars, 200 µm, were inserted using a micrometer and Fiji software.

*Confocal microscopy.* Spheroids were harvested and fixed in 4% paraformaldehyde. After a well was assembled on each slide to prevent spheroid morphology from being altered by the coverslip, spheroids were mounted on a slide with fluoromount G (Electron Microscopy Sciences, Hatfield, PA). An Olympus FV1000 confocal microscope with 488-, 559-, and 633-nm lasers was used to collect Z stack images of spheroids starting at the apex of the spheroid, proceeding every 10 µm until laser penetration faltered (~70 µm in depth). All images were taken using a 20× objective.

**SUPPLEMENTARY MATERIAL** **Figure S1.** Effect of serum.

## Supplementary Material

Supplementary Figure S1 Effect of serum.
