# Supplementary figures and images for: Multicellular Tumor Spheroids as a Model for Assessing Delivery of Oligonucleotides in Three Dimensions
Source: Mol Ther Nucleic Acids. 2014 Mar 11;3(3):e153–. doi: 10.1038/mtna.2014.5 (PMC4027982; doi:10.1038/mtna.2014.5)

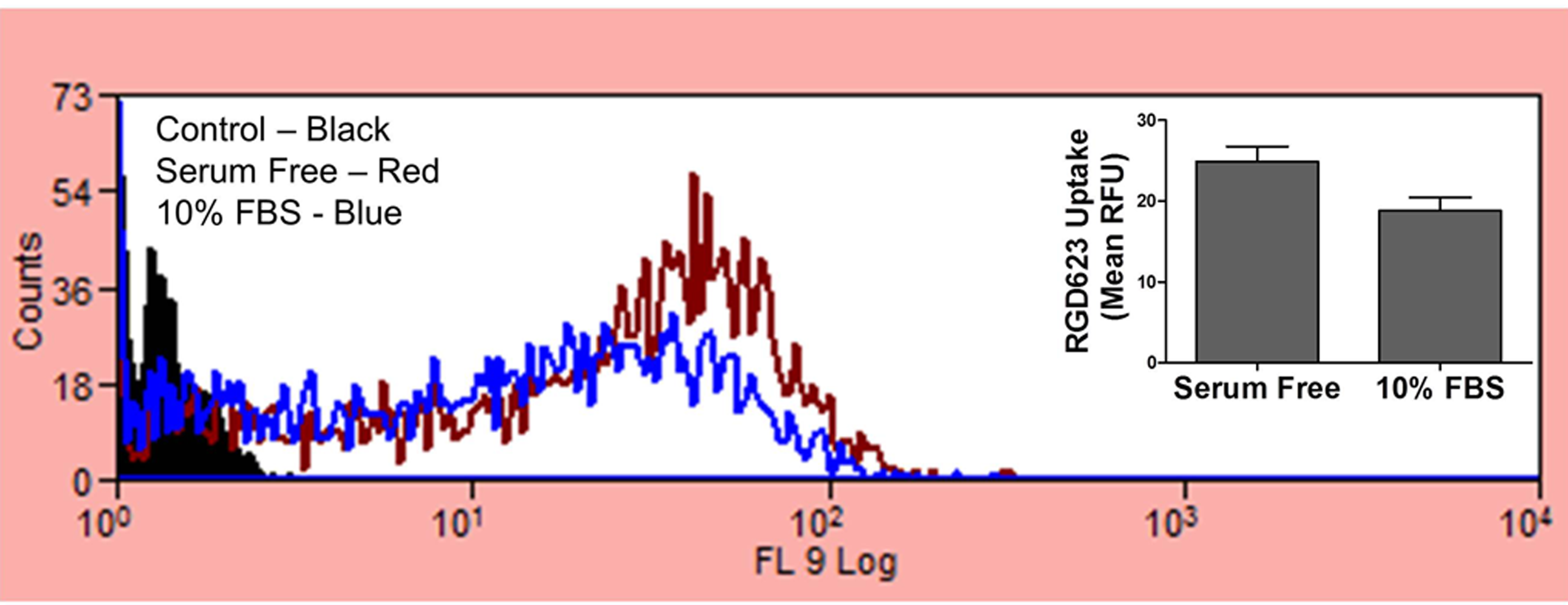

Supplement: Supplementary Figure S1 — Effect of serum. [file mtna20145x1.tiff]
